# The economic burden of health disparities related to socioeconomic status in Israel

**DOI:** 10.1186/s13584-019-0306-8

**Published:** 2019-05-27

**Authors:** Eran Politzer, Amir Shmueli, Shlomit Avni

**Affiliations:** 1 Harvard PhD Program in Health Policy (Economics), Harvard University, Cambridge, USA; 2 School of Public Health, Hebrew University-Hadassah, Jerusalem, Israel; 3 Health Inequalities section, Administration for Strategic and Economic Planning, Ministry of health, Jerusalem, Israel

**Keywords:** Health disparities, Socioeconomic status, Equity, Health care utilization, Sickness leave, Labor force participation, Disability allowance, Tax exemption, Health expenditures

## Abstract

**Background:**

Low socioeconomic status (SES) is often associated with excess morbidity and premature mortality. Such health disparities claim a steep economic cost: Possibly-preventable poor health outcomes harm societal welfare, impair the domestic product, and increase health care expenditures. We estimate the economic costs of health inequalities associated with socioeconomic status in Israel.

**Methods:**

The monetary cost of health inequalities is estimated relative to a counterfactual with a more equal outcome, in which the submedian SES group achieves the average health outcome of the above-median group. We use three SES measures: the socioeceonmic ranking of localities, individuals’ income, and individuals’ education level. We examine costs related to the often-worse health outcomes in submedian SES groups, mainly: The welfare and product loss from excess mortality, the product loss from excess morbidity among workers and working-age adults, the costs of excess medical care provided, and the excess government expenditure on disability benefits. We use data from the Central Bureau of Statistics’ (CBS) surveys and socio-health profile of localities, from the National Insurance Institute, from the Ministry of Health, and from the Israel Tax Authority. All costs are adjusted to 2014 terms.

**Results:**

The annual welfare loss due to higher mortality in socioeconomically submedian localities is estimated at about 1.1–3.1 billion USD. Excess absenteeism and joblessness occasioned by illness among low-income and poorly educated workers are associated with 1.4 billion USD in lost product every year. Low SES is associated with overuse of inpatient care and underuse of community care, with a net annual cost of about 80 million USD a year. The government bears additional cost of 450 million USD a year, mainly due to extra outlays for disability benefits. We estimate the total cost of the estimated health disparities at a sum equal to 0.7–1.6% of Israel’s GDP.

**Conclusions:**

Our estimates underline the substantial economic impact of SES-related health disparities in Israel. The descriptive evidence presented in this paper highlights possible benefits to the economy from policies that will improve health outcomes of low SES groups.

## Background

Health disparities have many determinants, including the political, social, and economic atmosphere in a country, the extent of social inequality, and living and environmental conditions, to name only a few [1]. We focus on health disparities related to inequalities in socioeconomic status (SES), that includes income, education, employment, and social status.

The social determinants of health have been extensively researched for many years. Specifically, evidence on the relation between SES and health outcomes such as mortality or morbidity is strong, long-standing, and observed in rich and poor countries alike. A brace of studies and data shows that higher income, higher education and better social status correlate with longer life expectancy and better health, and that this gradient exists at all levels of the socioeconomic scale [2–6]. The situation in Israel is no different.

Health disparities claim a human and social price along with a steep pecuniary economic cost. This is so, first, because poor health impairs personal welfare and premature death hurts the welfare of the deceased’s household and society at large. Second, poor health may harm the individual’s ability to acquire education, vitiate his or her working capacity, and impair other workers’ productivity. In addition, poor health has implications for the national health care expenditure and entails additional government outlays for disability support and benefits.

We examine how low socioeconomic status is related to additional morbidity and premature mortality in Israel, and assess the economic costs associated with these excess morbidity and mortality. To precisely estimate the cost of health impairment caused by socioeconomic gaps, one has to identify the causal effect of SES on health, accounting for possible reverse causality. While not attempting to undertake this task here, we present estimates that assign monetary values to *correlations* between SES and health. Much like similar studies in other countries [7–9], our estimates compare the existing inequalities to a counterfactual scenario, in which the socioeconomically “weak” population attains the average state of health of the “strong” population. To use the difference between the actual cost and that in the counterfactual, one has to essentially assume that the correlation observed originates entirely from the effect of the socioeconomic inequalities on health, and not from the reverese causality path. Since this is a strong assumption, that most likely doesn’t hold, the costs presented serve as only the upper bound of the actual costs. However, the costs presented consider the health disparities of the weak population only, whereas such inequalities exist more continuously along the “socioeconomic gradient,” including among people in better socioeconomic condition. If health inequalities on all rungs of the socioeconomic gradient were taken into consideration, the cost estimates found would most likely be higher.

Notably, while we use a more equal counterfactual in which the “weak” population’s health improves, we neither determine how this scenario can be realized nor estimate the cost of realizing it. Therefore, this study does not conduct a cost-benefit analysis for programs that narrow health inequalities. It may, however, serve as a first step in that direction because it estimates the benefit that may be gained by narrowing the gaps.

Three main studies [7–9] have attempted to estimate the economic burden of SES-related health inequality in the UK, the EU and the US, respectively. SES was measured by means of income [7], level of education [8], and race and ethnic origin [9]. The studies investigated three main groups of costs associated with health inequality: premature death and disability, loss of working days, and excess medical care. Table 1 details the estimated costs for each of these components. The studies’ methodologies are further reviewed in the next section. The studies that investigate SES-related health disparities in the UK and the US found an economic burden of 2% of GDP in these countries. The study concerning the European Union, in contrast, estimates the economic burden there at a much higher level—nearly 13% of GDP. Additional papers estimate only one of the cost components [10–14].

**Table 1:** Main studies estimating the economic burden of SES-related health inequality

Country (Year of estimation)	Socioeconomic index	Counterfactual scenario (more equal outcome)	Components of inequality-related economic burden: cost per year			Total
			Mortality and disabilities	Lost days of work	Excess medical care	
[7] UK (2010)	Income	The health level is made equal to that in neighborhoods with above the median income	Loss of 1.3 years of life per person: £2.2 billion loss of 2.5–3.8 disability-free years per person.	£31 billion	£5.5 billion	2.2% of GDP
[8] EU—25 countries (2004)	Education	The health level is made equal to that of the population half with higher schooling	Those dying lose 16 years of life on average: €700 billion. Lost years of life in good health: €280 billion	€141 billion	€177 billion (20% of total healthcare expenditure, 1.7% of GDP)	13% of GDP
[9] U.S. (2003–2006)	Race / ethnic origin	The health level is made equal to that of the healthiest ethnic group	Lost years of life: $239 billion	$13 billion	$57 billion	2% of GDP

## Methods

### Definition of SES-related health inequalities

We follow the “levelling up” approach and define the SES-related health inequalities by the gap between the real outcomes and a more equal counterfactual, in which the socioeconomically “weak” population attains the average state of health of the “strong” population. This approach is used in the three papers that estimate the economic burden of SES-related health inequality in the UK, the EU, and the US [7–9], and is closely related to the epidemiological measure of Population-Atrributable Risk (PAR) [8]. We mark the line between the “weak” and “strong” subpopulations at the median of our chosen SES measure, following the UK and EU papers [7, 8], that also use such a simple 50% dichotomy between low and high SES (the EU paper [8] also examines a second counterfactual, in which the separating line passes at 90%). The decision regarding the line separating between weak and strong, and the derived definition of the counterfactual, is rather arbitrary. With a higher line, the cost of inequalities is calculated for a larger group with a higher SES, and the counterfactual becomes more ambitious, up to complete equality when only the top individual is considered “strong”. This extreme case will certainly produce an overestimation of costs, since part of the observed correlation between SES and health is due to the effect of health on SES, and not the otherway around. Setting the line at the median ignores health inequalities within the strong half of the population, but leads to a conservative estimate of costs, that may account for some reverse causality.

### Measures of socioeconomic status

We use three different measures to define the socioeconomic status, mostly depending on practical reasons, i.e. the availability of the SES measures alongside the examined health outcomes in our data sources. First, we use the socioeceonmic ranking of localities, which is available along data on mortality, hospital discharges, and payment of disability allowances. The SES of geographical units is used also in the study from the UK [7], that exploit data on the average income in UK neighborhoods. However, the average Israeli locality with data on mortality holds 51,000 individuals on average - much larger than the average neighborhood examined in the UK (with 7000 residents). The examination of large localities ignores possible heterogeneity within the locality, between strong-SES and weak-SES neighborhoods. This may bias down our cost estimation relative to the neighborhood-estimate in the UK study [7]. The second SES measure we use is the individual’s income, specifically the income per standard person in the individual’s household, which is available along data on absenteeism from work and along data on the use of community care. Using individuals’ SES helps eliminating possible biases from heterogeneity within larger units of analysis such as neighborhoods or localities. However, analysis of income and health outcomes from the same period may aggravate estimation biases due to reverese causality, as individuals’ health shocks may lead to temporary changes in income. The third SES measure we use is individuals’ education level. This measure attenuates possible biases from reverse causality, as adults’ education level is mostly established before their health outcome is observed, and so will be less sensitive to temporary health shocks. However, education may be a less accurate measure of SES than the actual income, as SES may vary greatly within each education level. We use the education level as an SES measure for non-employed individuals, where income data is unavailable.

### The estimated costs

Health is valuable both as an invstement good that increases human capital and production, and as consumption good that is itself a source for social welfare. Like the main parallel studies [7–9], we estimate costs of SES-related health inequalities in both dimensions. As an investment good, we examine the correlation of low SES with increased mortality and morbidity that lead to premature death before retirement, absenteeism, and non-participation in the labor force. We don’t estimate the costs of non-participation due to informal care given to a sick relative – a cost area reviewed in the UK study [7]. As a consumption good, we estimate the welfare loss from premature deaths at all ages. Due to lack of data, we don’t estimate the welfare loss from excess morbidity that is examined in [7].

In addition to these variables, we estimate separately the direct costs to the health care system from excess medical care provided. We also estimate the costs incurred by the government on disability benefits and the tax exemption to the disabled. From a macroeconomic perspective, disability benefits are transfer payments and have no direct effect on the GDP. From the government’s standpoint, however, they are an expenditure that could be put to other use and must be funded through possibly distorting taxes. Within the government budget, we also estimate the reduced spending on old-age benefits due to the excess mortality. Mostly because of data limitations, we don’t examine the cost of excess unemployment benefits [7, 8], nor the loss of tax revenue due to increased worklessness [7] or premature death of workers. Table 2 describes the estimated cost areas related to each health outcome. It details the SES measure we use for the estimation, the prices by which outcomes are monetized, and the data we exploit.

**Table 2:** Examined health outcomes and estimated costs

Health outcome	Definition of the low-SES group	The estimated cost	Prices used for monetization	Data sources
1. Mortality	Localities with a submedian socioeconomic index	1a. Product loss due to premature death of workers and deaths before the working age	Workers’ wages	CBS socio-health profile of localities for 2005–2009; CBS profile of municipal authorities for 2009 and for 2010
		1b. Welfare loss due to excess mortality	The value of statistical life: The value used by the Ministry of Transport, or 3 times GDP	
2. Morbidity	Individuals with a submedian net household income per standard person	2a. Product loss due to illness-related absence from work	Labor wages of submedian workers (absentees or non-absentees)	CBS social surveys (2010, 2012)
	Individuals with secondary schooling or less	2b. Product loss due to higher rates of non- employment due to illness	Labor wages of workers with secondary schooling or less	
3. Medical care	Localities with a submedian socioeconomic index	3a. Excess inpatient care (measured by excess hospital discharges)	MoH price per inpatient day X average length of hospitalization	MoH data on hospital discharges rates; MoH price schedule (2014)
	Individuals with a submedian net household income per standard person	3b. *Savings* on community care (due to underuse of services)	MoH service prices	CBS’s matched Health Survey (2009) and Income Survey (2010); MoH price schedule (2014)
4. Disability	Localities with a submedian socioeconomic index	4a. Excess government’s expenditure on disability benefits	Rates of disability benefits	NII data on disability benefit payments
	Individuals with submedian labor income	4b. Excess cost of the government’s income tax exemption for to the disabled and the blind	Workers’ tax exemption	The Israel Tax Authority’s sample of employees
5. Old-age survival	Localities with a submedian socioeconomic index	Government’s *savings* on old-age benefits (due to premature mortality)	Rates of old-age benefits	NII data on old-age and survivors’ benefits
6. Other	–	Cost of MoH programs to narrow SES-related health inequalities		MoH data

*Abbreviations: GDP* Gross Domestic Product, *CBS* The Central Bureau of Statistics, *MoH* The Ministry of Health, *NII* The National Insurance Institute

**First**, to estimate the cost of premature death we examine age- and gender-specific mortality gaps among localities with different socioeconomic ranking. The analysis is based on age-standardized mortality data in Israel’s 109 large localities with more than 10,000 residents, where 85% of the country’s population reside (we extrapolate the costs to the whole population). The data appear in the socio-health profile of localities in Israel for 2005–2009 (the latest available years), published by CBS and the Ministry of Health [15]. We also use data from the CBS profile of municipal authorities in Israel in 2009 and 2010. The CBS socioeconomic index is based on sixteen variables related to demography, education, employment and retirement, and standard of living (including per-capita income). We calculate the population-weighted median of the index, and divide the localities into two groups – above and below the median;

Within the human-capital approach, the cost of the excess mortality in socioeconomically submedian localities is assesed based on the discounted value of the lost income in the labor market from the time of death to the retirement age.^1^ We assume that the employment rate of the deceased would have been equal to the average employment rate in each locality,^2^ and their wage equal to the average wage for their gender in their locality. If the wage of the marginal deceased worker reflects the value of her marginal output, the sum of lost wage payments should also mirror the loss of national product due to premature death (ignoring general equilibrium considerations). The calculated cost is adjusted to 2014 terms by the growth rate of the population in each below-median locality and the national growth rate of the average wage.

Under the welfare approach, the value of a year of life does not depend solely on labor income, but also expresses an estimate of the total welfare flowing from the continuation of life. To perform the calculations in this study, we use two conventional valuations of a statistical year of life: first—a value derived from the procedure used by the Ministry of Transport in the evaluation of transportation projects. The procedure sets the life of a traffic fatality at 1.7 million USD (approximatly 6.1 million NIS at an exchange rate of 3.577 – the average exchange rate in 2014). Dividing this by average years of life lost per fatality (42 years), a value of 41,930 USD per statistical year of life is obtained. The second value emerges from the literature [16], where this value is sometimes estimated at a three times per-capita GDP—112,000 USD in 2014 terms in Israel. For a discussion of the approaches to value health and life years see [8]. We measure the years lost between the time of actual death to the time of death predicted by the conditional life expectancy for the deceased’s age and gender in the entire population [17, 18]. For the calculation, we assume that those who die after age seventy-five (4% of the population) lose no years of life. The cost was adjusted to 2014 terms by the growth rates of the population in each below-median locality.

The **second** variable we examine is the cost of days of work lost due to SES-related excess morbidity. We use data on individuals who were surveyed in the CBS Social Survey (2010) [19] and reported being employed but having missed work in the previous month due to illness. We divide the employed into two quantiles by net income per standard person in their household, and compare the probabilities of missing some work due to illness, and the length of absence. The cost of excess illness-related absence is first estimated using individuals’ labor wages.^3^ Noting that workers with some absence receive lower wages, we also estimate the cost using the wages of workers without any illness-related absence during the same month. For workers reporting missing only part of the day, we assumed that partial absence means the loss of one-third of a work day (the survey offers no data on the number of hours missed). Costs were adjusted to 2014 terms by the growth in national average wage and the change in the working population.

In addition to that, we used the Social Survey data to examine, by individuals’ education, the share of people who do not work at all due to illness. We compared individuals with secondary schooling or less, to those with post-secondary or academic education. We calculate the cost of higher rates of non-working among poorly educated persons on the assumption that had these individuals been working, their wages would have resembled those of other poorly educated persons. In the counterfactual, the share of non-working persons among those with little education is identical to that among the well educated in the same age and gender group.

**Third**, we investigate the costs of excess medical care due to SES-related poor health. To investigate inpatient expenditures, we use the Ministry of Health locality-level data on hospital discharges rates in 241 localities (that have a population of 2000 or more) and in regional-council jurisdictions, where altogether 99% of the Israeli population lives [15]. We examine the age-standardized discharge rate per 1000 residents, in localities with socioeconomic index below and above the median. The cost of the additional hospital discharges in weak localities is monetized using the average length of hospitalization - 4 days (we use the national average due to lack of data on the length of stay by locality), and the MoH regulated prices per inpatient day in 2014. To estimate inequalities in the use of community-based healthcare services, we use the matched data from the 2009 Health Survey [19] and the 2010 CBS Income Survey [20]. We compare use of health services by individuals above and below the median standard per-capita household income, in each age and gender group. We examine visits to primary physicians (family-practice physician, pediatrician, internist, obstetrician-gynecologist), visits to secondary physicians (specialists other than primary physicians), visits to paramedical professionals (physical therapists, occupational therapists, communication clinicians, dietitians), and use of MRI scans (other than those entailing hospitalization). The *saving* in health expenditures, due to low use by lower-income individuals, is estimated using the 2014 MoH’s prices.

**Fourth**, we use 2014 data from the Israel National Insurance Institute [21, 22] to examine the costs to the government from excess disability benefit payments in socioeconomically weak localities. The data describe disability benefit payouts in 194 municipal and local-council jurisdictions (that have populations of 2000+) and in fifty-two regional-council jurisdictions. Three disability benefits are examined: general disability (for persons aged eighteen up to retirement age), special services, and children with disability (up to age eighteen). We divided the localities into above- and below-median quantiles based on each locality’s socioeconomic index.

In addition to that, we examine the cost to the government from the income tax exemption to the (severely) disabled and the blind. We exploit administrative 10-year panel data from the Israel Tax Authority [23], that includes an annual sample of 10% of all employees. We estimate the number of recipients and the total cost of the tax exemption. To estimate the excess cost for low-income employees we examine individuals that received the disability exemption in 2013 but not five years earlier, in 2008. Assuming that these individuals suffered an acute event that created the disability and qualified them for the exemption during those five years, we use their 2008 income decile to classify their pre-disability SES. We then calculate the probability of receiving the exemption in 2013, above and below the median income in 2008, and use it to estimate the cost of SES-related exemption take-up.

**Fifth**, we examine the government’s savings on old-age benefits due to premature mortality in low-SES localities. This cost area is not examined in parallel studies, but since old-age allowances constitute a large government expenditure, it is important to understand the effect of the counterfactual on these benefits. To estimate the savings, we used 2014 data from the National Insurance Institute on average old-age and survivors’ benefits in each locality. For a deceased in each age and gender group, we calculate the discounted value of the old-age benefits that the person would have received, from retirement age, or from actual death if it occurs after retirement, until her predicted death according to the life expectancy at the age of death.

**Finally**, as the sixth and last cost item, we reviewe the sums spent by the Ministry of Health for the specific purpose of narrowing health inequality [24]. Thusfar, we estimated the inequality patterns mostly on using data from 2005 to 2010, before the Ministry of Health launched a program to narrow the gaps (in 2010/11). Consequently, government expenditure on narrowing the disparities may be seen as a *result* of the disparities, and one may construe total government expenditure on narrowing health disparities in 2011–2015 as part of the economic costs calculated above. We estimate the Israeli government expenses on initiatives to reduce health disparities based on reports by various government units from the Israeli MoH, assembeled by the strategic and economic planning division in the MoH. In calculating government expenditures we include only actions relevant to SES disparities.

## Results

### The cost of SES-related premature mortality in Israel

**Fig. 1:**
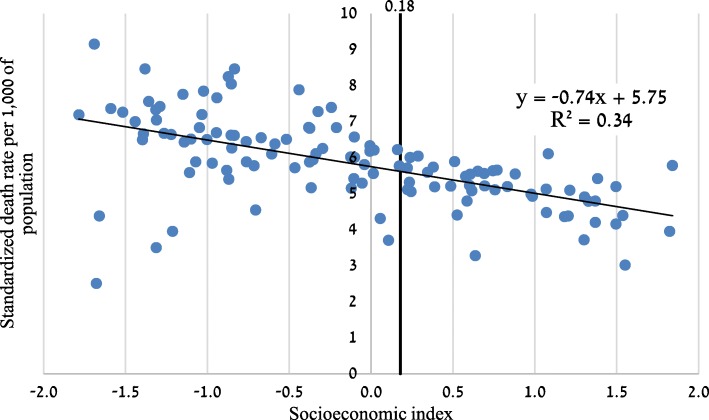
Standardized death rate per 1000 of population and socioeconomic index in localities with 10,000+ population. Legend: Median = vertical line

There is a negative correlation between each locality’s standardized mortality rate and its socioeconomic index (Fig. 1). The characteristics of localities above and below the median index are described in Table 3.

**Table 3:** Selected characteristics of localities in each socioeconomic index quintile (above/below median), 2005–2009 average (unless noted otherwise)

	Quintile	
	Below median	Above median
Total population (,000)	3230	2866
Localities (N)	73	46
Avg. locality population (,000)	44.3	62.3
Avg. socioeconomic index (weighted by locality population)	−0.49	0.80
Share of 0–24 age group	49%	36%
Share of 55+ age group	15%	23%
Share of Jews (2010)	62%	93%
Share of men	49.7	48.6
Avg. wage of men—weighted avg. (2009), USD	1780	2885
Avg. wage of women—weighted avg. (2009), USD	1204	1782
Employment rate (2009)	70%	84%
Age-standardized death rate per 1000 of population	5.85	5.27
Age-standardized death rate per 1000 of population, men only	6.70	6.13
Age-standardized death rate per 1000 of population, women only	5.16	4.58

**Fig. 2:**
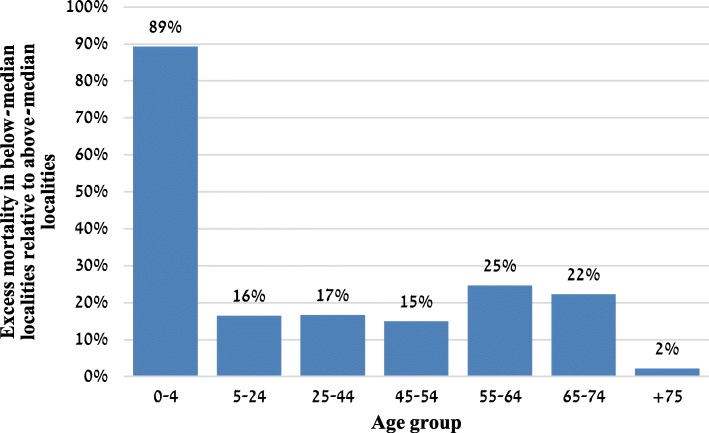
Excess mortality in submedian localities relative to above-median localities, by age group (pct)

We find that mortality in localities below the median socioeconomic index exceeds that in above-median localities in all age groups (Fig. 2). For example, the annual mortality rate per 1000 of population aged 55–64 in submedian localities is higher by 1.4 deaths than in above-median localities (25% higher), and the under-five mortality rate is higher by 0.6 deaths per 1000 children at that age (89% higher).

### Loss of national product due to premature mortality—the human-capital approach

**Fig. 3:**
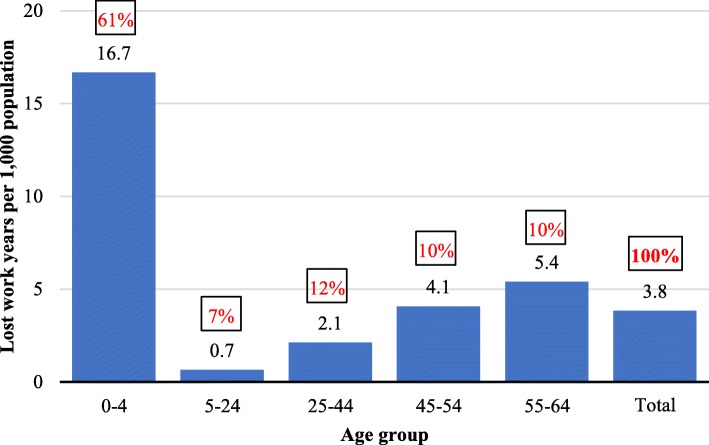
Lost work-years per 1000 of population in each age group, submedian localities. Legend: In a box - contribution of the age group to total lost years (pct)

Annual excess mortality in submedian localities, relative to the counterfactual (i.e. the average in above-median localities), sums up to a loss of some 11,000 work-years in the economy—about 3.8 work-years per 1000 working-age persons in submedian localities. Most of the gap (64%) owes its origin to males who die prematurely. Divided by age groups, 61% of the lost work-years come from excess under-five mortality (who lose their whole working life), and 20% from excess mortality in ages 45–64 (Fig. 3).

The discounted value of wage payments that were lost from the date of death to retirement is calculated for each submedian locality and each age and gender group. In 2014 terms, the lost wage payments due to excess mortality add up to 0.14 billion USD.

### The social cost of premature death—the welfare approach

**Fig. 4:**
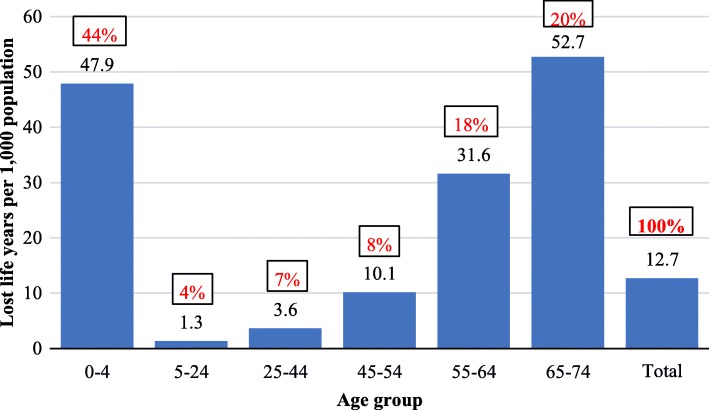
Lost years of life per 1000 persons in each age group, submedian population. Legend: In a box - contribution of the age group to total lost years (pct)

We find that around 41,000 years of life are lost each year due to excess mortality among the submedian population—12.7 per year per 1000 of this population. By age groups (see also Fig. 4), the lost years of life trace mainly to excess mortality in the 0–4 age group (44%) and the 55–74 cohort (38%). In 2014 terms, the cost of excess submedian mortality is estimated at 1.09 billion USD per year, when we use the value of a year of life derived from the Ministry of Transport’s procedure. The cost rises to 2.9 billion USD**,** with a year of life valued at three times per-capita GDP in 2014.

### Cost of workdays lost due to SES-related excess morbidity

#### Absenteeism due to illness

**Fig. 5:**
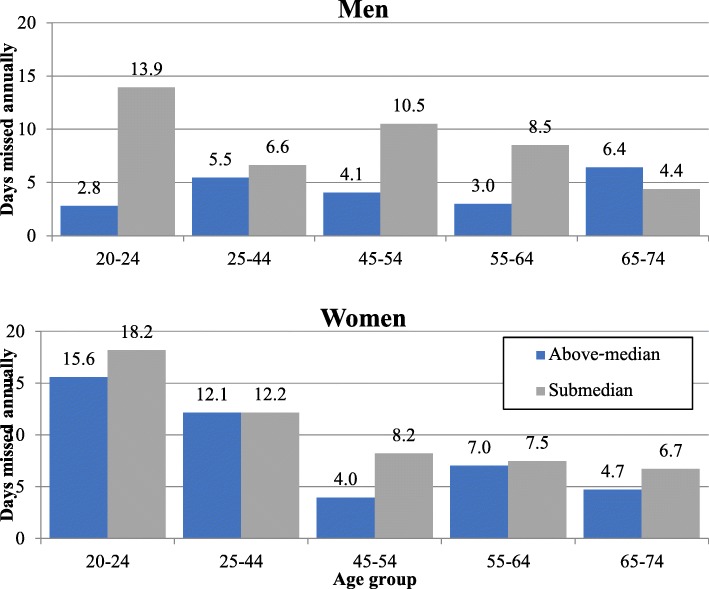
Work days missed due to illness. Note: annual average per worker, by medians of standard per-capita income in workers’ household, parsed by gender and age groups

The share of workers missing full days of work due to illness was higher among workers with submedian income than among workers above the median—17.3% as against 15.4% (Table 4). Absentees’ average length of absence in the previous month was also longer in the submedian group—4.6 days as against 3.7 among those above the median. Figure 5 shows the derived annual average of days missed per worker in each income quantile, parsed by gender and age groups. Focusing on workers of prime working age (25–64), it is evident that the disparities in absenteeism caused by illness are especially wide in the 45–54 cohort and that they remain large among men aged 55–64. In 2014 terms, the total cost of excess absenteeism due to illness in the submedian income group adds up to 0.34 billion USD per year. If the calculation uses the higher average wage of workers who didn’t miss any working day, the total cost of excess absenteeism climbs to 0.45 billion USD.

**Table 4:** Characteristics of workers absent due to illness, by quantiltes (above/below the median income), 2010

	Working individuals (N)	Missed full day of work (N)	Percent of workers absent	Avg. absence in month per absentee (days)	Avg. wage of absentees (2010 USD)	Avg. wage of non-absent individuals (2010 USD)
Submedian	1,139,019	197,348	17.3%	4.57	1476	1661
Above-median	1,080,272	165,831	15.4%	3.67	3066	3437

In the 2010 Social Survey [19], 7% of workers reported missing part of a workday due to illness. The proportion of partial absentees is slightly higher in the submedian group (7.4%) than in the above-median group (6.8%); the number of partial-absence days is also higher in the submedian group (Table 5). We calculate the cost of partial absenteeism against the counterfactual, in which the same absentee rate prevails among members of the same age and gender groups in both quantiles. The excess cost in the submedian group is 31 million USD per year.

**Table 5:** Characteristics of workers absent during part of a workday due to illness, by quantiles (above/below the median income), 2010

	Working individuals (N)	Missed partial day of work (N)	Pct. Partially absent among all workers	Avg. days of partial absence per absent person during month	Avg. wage of partially absent individuals (USD)	Avg. wage individuals not reporting partial absence (USD)
Submedian	1,136,832	84,013	7.4%	3.59	1487	1642
Above-median	1,079,683	73,866	6.8%	3.28	3303	3387

#### Unemployment or nonparticipation in the labor force due to illness

**Fig. 6:**
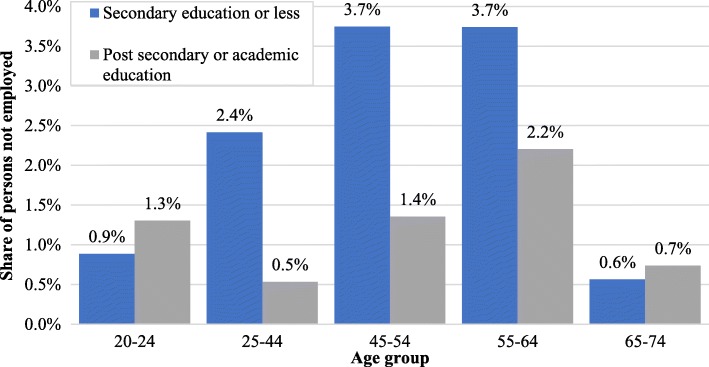
Share of persons not employed due to illness, by age groups

According to the CBS Social Survey (2012) [20], 1.7% of adults do not work for reason of illness (i.e., physical limitation, disability, or protracted illness). About one-fourth of them are unemployed (i.e. actively searching for work); the others are entirely out of the labor force. The share of non-workers due to illness among those with secondary or less education - 2.3% - is twice as great as that among those with post-secondary or academic schooling - 1.1% (see Table 6). The difference is particulary large among those aged 25–54 (Fig. 6). In comparison with the counterfactual and in 2014 terms, the cost of excess non-working due to illness among persons with secondary education or less is 0.92 billion USD per year.

**Table 6:** Adults (age 20+) not working due to illness, by levels of education (2012)

	Individuals in population	Unemployed due to illness	Non-participants in labor force due to illness	Total not working due to illness	Share of those not working due to illness
Secondary education or less	2,524,766	12,441	44,585	57,026	2.3%
Post-secondary or academic education	2,121,066	6202	16,174	22,917	1.1%

### Cost of excess medical care due to SES-related poor health

#### Inpatient care

**Fig. 7:**
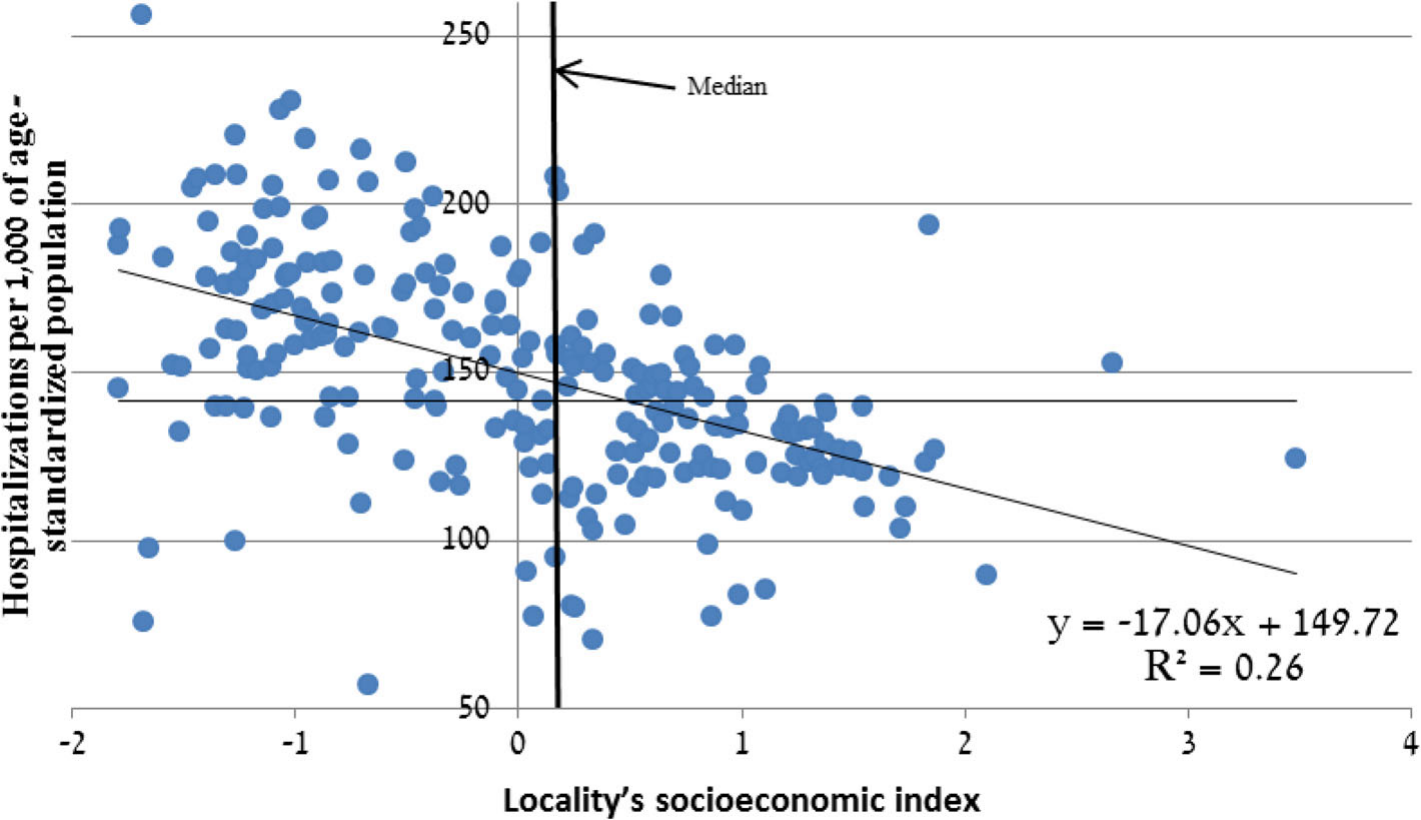
Age-standardized hospital discharges per 1000 of population by socioeconomic-index in localities exceeding 2000, 2005–2009 average. Legend: Median = vertical line

We find a negative correlation between the number of hospital discharges in each locality and the locality’s socioeconomic index (Fig. 7). In submedian localities, there are 156.2 age-standardized discharges annualy per 1000 of population—10% higher than in localities above the median (i.e. 14.5 additional hospital discharges per 1000 of population per year).

Excess hospital discharges in weak localities account for 5% of total annual discharges (in 2014—65,000 discharges out of 1,300,000 altogether). The cost of excess hospital discharges is 176 million USD per year (in 2014 terms).

The estimate remains identical when omitting several outlier localities (mostly populated by the ultra-Orthodox), that have low hospitalization rates even though they rank low on the socioeconomic index. To account for differences in hospital availability and accessibility, we divide the localities to two groups by their peripherality index (CBS) [25], or by the number of hospital beds in the locality’s district [26], and then recalculate the SES-related gaps. These calculations (available from the authors) lead to similar cost estimates (140 to 201 million USD).

#### Community-based care

Use of primary physicians’ services is similar on average among individuals above and below the median income (Table 7). However, those in the above-median group make 30% more visits to specialist secondary physicians, visit 60% more paramedical professionals, and undergo 90% more MRI scans.

**Table 7:** Personal characteristics and average annual use of community-based health care services in each income quantile (below/above median), 2009

	Quantile	
	Submedian	Above-median
Observations in survey	13,502	11,093
Individuals in population	3,342,155	2,716,041
Avg. size of individuals’ households	3.75	3.13
Net income per standard adult in individual’s household (USD)	590	1849
Share of children and young adults (up to age 24) in households	51%	36%
Share of persons aged 65+	10%	10%
Share of persons with chronic or protracted illness^a^	19%	24%
Share of persons with disabilities^b^	4.3%	3.2%
Visits to a doctor (avg. per individual)	6.70	7.07
Thereof: to a primary physician	5.17	5.10
Thereof: to a family-practice physician	3.68	3.74
to a secondary physician	1.53	1.97
Visits to paramedical professionals	0.73	1.16
MRI scans (not entailing hospitalization) per 1000 persons	10.86	20.74

^a^The share of persons with at least one of the following illnesses or conditions: hypertension, heart attack or myocardial infarction, other heart disease, stroke, diabetes, asthma, chronic pulmonary disease, chronic digestive condition, cancer, depression, or anxiety

^b^The survey participants were asked if they had disabilities in moving about outside their homes and in ascending and descending stairs and whether they could, unassisted, get dressed, bathe, eat, sit down and stand up, and get into and get out of bed

In monetary terms, the less prevalent use of community-based services by the poor *saves* on expenses, relative to the counterfactual. The extent of saving on services that we examined, after summing the gaps for each gender in each age group, comes to 81 million USD per year (in 2014 terms).

To account for differences in access to community-based services, we divide the individuals to two groups, by the ratio of community-based doctors per 1000 of population in the individuals’ district of residence [27]. When recalculating the income-related gaps, we find that the differences in the number of uses between the income quantiles are lower in areas with higher accessibility. The estimated saving in this calculation (available from the authors) is 50% lower (43 million USD per year). In any case, the saving falls short of the excess expenditure on hospitalization services (176 million USD).

### Disability-related general-government expenditure outside the health sector

#### Disability benefits (National Insurance Institute)

A higher share of the population receives disability benefits in submedian localities than in above median localities (Table 8). The rate is one-fourth higher for general-disability and special-services benefits even though the share of the relevant population—those aged 20–64—is 10% lower than in the above-median group. Receipt of child-disability benefits is 40% higher in submedian localities, mostly due to a higher share of childrens in these localities. As for the size of the benefit (that depends on the severity of the disability), the gaps between the quantiles are not large. Accounting for the share of recipients and the level of the benefit, total per-capita payout of disability benefits is 30% greater in submedian localities than in those above the median.

**Table 8:** Locality characteristics and payout of disability benefits, by quantiles above and below socioeconomic index median (2014)

	Quantile	
	Submedian	Above- median
Localities	138	108
Population size	4,210,253	4,000,026
Avg. socioeconomic index (weighted)	−0.53	0.82
Demographic composition of quantile (pct.):		
Women	50.1%	50.8%
Age 0–19	40.9%	30.5%
Age 20–44	33.6%	34.8%
Age 44–64	16.9%	21.3%
Age 65+	8.6%	13.4%
Share of benefit recipients (pct.)		
General disability	3.04%	2.41%
Special services	0.66%	0.52%
Child with disability	0.60%	0.43%
Avg. monthly benefit of benefit recipient (USD):		
General disability	834	768
Special services	812	842
Child with disability	661	667
Total disability payout per capita in quantile (USD)	34	26

We estimate the excess payout of disability benefits at 0.42 USD billion due to higher eligibility for these benefits in localities below the median socioeconomic index. Some 75% of this sum originates in excess payments of general-disability benefits. The excess payments amount to 13% of the total payout of benefits by the National Insurance Institute Disability Division in 2014.

#### Tax exemption for persons with disabilities and the blind

We estimate that some 15,000 employees received the exemption in 2013. Half of them were relatively long-time eligibles who had also received this exemption in 2010. The average labor income of those exempt in 2013 was 2750 USD per month; their median income was 1490 USD. The tax expenditure resulting from the exemption in 2013 was 138 USD million. Examining the data by income deciles (of employees’ current labor income), we find that 58% of the cost of the tax expenditure grows from disabled workers in the two highest income deciles and 30% from the lowest decile. Using the Tax Authority’s panel data, we estimate for each decile of the 2008 income distribution, the probability of receiving a disability exemption five years later. We find that the likelihood of receiving a disability exemption is only slightly higher among those above the median income (0.168%) than among those below it (0.161%). Therefore, in the counterfactual scenario, i.e. if the likelihood of exercising the disability exemption is the same above and below the median, the effect on state revenues is negligible - The state incurs an additional cumulative tax expenditure of only 1.1 USD million. The results are similar for a gap of 6 or 7 years, instead of 5 years.

### Saving on old-age-benefit expenditure due to premature mortality

Excess mortality in low-SES localities reduces the government’s expenditure on payments to the elder population. Relative to the counterfactual, excess mortality in submedian localities saves the state 126 USD million per year (discounted value in 2014 terms). This sum is equal to 1.7% of total payouts by the NII’s Old-Age and Survivors Division in 2014.

### Cost of the Ministry of Health’s inequality-mitigation intervention programs

Different countries tackle health inequality in different ways. A 2013 study [28] found that most European Union countries (88%) have no national strategy for tackling health inequality even though they do have national-level interventions that may be considered valuable in narrowing inequality indirectly (i.e., interventions that are derived from a general policy on improving health or welfare, or focusing on vulnerable population groups that suffer from health inequality).

Israel has a dedicated national program for the mitigation of health disparities. In late 2010, the Ministry of Health publicized its objectives as part of its comprehensive work plan for 2011–2014, known as “Pillars of Fire”. In 2015, in a revised version of “Pillars of Fire”, the objective of narrowing inequality was paired with the promotion of public health. The activities spearheaded by the Ministry of Health included, among others, the following [29]: lowering copayments for services and medicines that are covered by national health insurance, such as abolition of the child and mother centers fee, significantly increasing state participation in the costs of rehabilitation services (to 75%), giving the elderly (75+) a 10% discount on medicines, lowering the maximum copay for medicines for chronically ill elderly and recipients of income assurance benefit; expanding national health insurance to cover additional essential services: preservative and preventive dental care for children up to age fourteen, and vaccinations; eliminating linguistic and cultural impediments to access to healthcare services—setting up a medical translation call center, developing tools for training on the topic, and training cultural supervisors and instructors; expanding the supply of human resources in the periphery and among minority groups by creating grants and wage bonuses for doctors and nurses in the geographical periphery, grants and cover of tuition for Bedouin women nursing students; prioritization of the periphery in developing infrastructure and allocating technologies—investing in building and development, establishing urgent medicine centers, and adding MRI machines; incentivizing HMOs to develop additional activities to narrow gaps by offering conditional grants; adding geographical variables to the risk-adjustment formula by which the HMOs are payed; and setting up a center on inequality to monitor activities and publish and disseminate the knowledge amassed.

In 2011, the first year of the intensified activity, 0.31 USD billion was spent on the ministry’s program for mitigation of health inequality. The expenditure increased gently over the years, to 0.39 USD billion in 2015. Total government expenditure on narrowing healthcare inequality during the years of the program, 2011–2015, was 1.73 USD billion—0.34 USD billion on an annual average [19]. Most of it was spent for purposes related to system availability (deployment of services and staff), access (economic and provision of information), and developing the system’s ability to cope with inequality (control, supervision, incentivization, training, etc.). Aproximatly 0.64 USD billion (0.14 USD on an annual average) of these expenses directly relate to SES disparities (as opposed to expenses due to cultural or georgraphical disparities).

### Summary of the costs

Table 9 tallies the economic burden of health inequality associated with socioeconomic status in Israel in 2014 terms (beside the negligible cost of the tax exemption for the disabled). Altogether, the economic burden of SES-related health disparities is 2.07 USD billion (0.7% of Israel’s GDP), when the costs of premature mortality and excess morbidity are calculated following the human-capital approach. When the welfare approach is used to calculate the cost of premature mortality, the economic burden climbs to 3.02–4.86 USD billion (1–1.6% of of Israel’s GDP), depending on the value attached to a statistical life year. Due to lack of data, this figure includes only the loss of product due to excess morbidity, and not the total welfare loss that would have been more suitable for the welfare approach. It should be noted again that both sums include costs that affect the GDP together with costs that are considered transfers within the economy.

**Table 9:** Breakdown of the economic burden of SES-related health inequality, 2014 terms

Component of burden	Cost (USD billion)	Remarks
*Welfare approach (health as a consumption good)*		
1. Lost welfare due to premature mortality (welfare approach)	1.09–2.94	
*Human-capital approach (health as an investment good)*		
2. Lost product due to premature mortality	0.14	
3. Lost product due to excess morbidity	1.4	
Total product loss (2 + 3)	1.54	
*Costs to the health care system*		
4. Excess inpatient care	0.17	5% of all annual hospital discharges
5. Reduced community care	−0.08	
Total excess medical care (4 + 5)	0.08	1% of public healthcare expenditure
*Additional costs to the government*		
6. Extra outlay for disability benefits	0.42	13% of disability payments
7. Savings on old-age benefits	−0.11	
8. Ministry of Health’s expenditure on mitigating health inequality	0.14	
Total additional costs to the government (6 + 7 + 8)	0.45	
*Total costs*		
Total costs, using the human-capital approach (2 + 3 + 4 + 5 + 6 + 7 + 8)	2.07	0.7% of of Israel’s GDP
Total costs, using the welfare approach (1 + 3 + 4 + 5 + 6 + 7 + 8)	3.02–4.86	1–1.6% of Israel’s GDP

## Discussion and conclusions

The findings indicate that health inequality associated with socioeconomic status imposes a significant economic burden on the State of Israel. Israel is not unique in this respect: similar findings from the UK, the European Union, and the United States point to even heavier burdens in terms of percent of GDP. Naturally, the national estimates of the burden of SES-related health inequality depend crucially on the method chosen (the unit of analysis, the operational definition of equality), data availability, the social value of life years and years lost, the valuation of productivity loss, and other computational assumptions (e.g. the discount factor used nationally). Consequently, the estimates of the burden are aimed to provide an order of magnitude rather than accurate figures. The main contribution of this paper is the calculation of the burden of health inequality in Israel, stressing to the policy makers the waste and the avoidable cost associated with SES-related health inequality in Israel. While, as mentioned above, international comparisons of the burden are problematic, the similar order of magnitude resulting from the UK and US studies [7, 9], in terms of GDP share, provides some support to our calculation.

When calculating the cost of lost working days due to illness, we find that workers who miss work have lower wages both above and below the median income. That is, the wages of illness-related absentees were 11% lower than those of workers who did not report such absence (Table 3). The reason may be that some illness-related absenteeism was not covered by paid sick leave; therefore, the wages of these workers in the month of their absence were lower than their regular wages. Another possible explanation is that the excess morbidity of absentees may impair these workers’ skills and productivity in the long term and lead to lower wages. Such impairment may prompt ill individuals to take lower-paying / lower-productivity jobs ab initio.

The calculation of the cost of lost working days has some limitations. First, we assumed that a worker’s wage reflects her marginal output – implicitly assuming that a day of absence impairs production at the value of the daily wage. However, absentees and their colleagues may compensate for absences. The more common such behavior is, the less product is lost to absenteeism; in this case, absenteeism would reflect nothing more than temporary volatility in labor productivity. Therefore, the estimate obtained may be an upper bound of the harm caused by illness-related absenteeism. Second, workers who wish or need to skip work and cannot use paid vacation days for this purpose may explain their absence as due to illness and thus use sick leave as a substitute for vacation days. However, the Social Survey data [19] suggest that absentees in both quantiles are entitled to similar amounts of paid vacation—around nineteen days per year. Furthermore, according to the survey, the share of absentees entitled to sick leave from the first day is smaller in the submedian group (55%) than in the above-median group (61%). This disparity, contrarily, weakens the incentive among lower-quantile workers to take short sick absences (which sometimes serve in lieu of vacation days). Third, we notice that the division to quantiles (above/below the median) uses income from the same year in which the health outcome (absenteeism) is measured. Therefore, the outcome may be affected by cases of reverse causality, in which high-income people fall into the submedian cohort due to an illness that impairs their income. Such cases may erroneously widen the measured health inequality between the income quantiles, biasing the estimated economic burden upward. Estimation based on individuals’ education, as is done for people outside of the work force, attenuates this concern, mainly in regard to health outcomes that are realized only years after education is acquired. When we repeated the calculation using individuals’ education instead of their income (calculation not shown), the cost of the inequality remained similar.

We find that low-income people use less community-based medical services than the high-income. The disparity is abetted by sometimes-limited access to secondary medicine and use of inpatient care instead of community-based services by the poor. Given that hospital care is more expensive than community-based care and is usually needed in later stages of an illness, the mix of services that the poor consume points to an inefficiency and, possibly, belated treatment of evolving illnesses. Beyond this, since the Health Survey examines uses that are both publicly and privately funded, the lower incidence of private insurance among low-income persons may further affect their access to specialists, paramedical professionals, and advanced tests such as MRI scans.

In the levelling up counterfactual, low-SES people will use more community care and less inpatient care, and the total annual cost of care will be reduced. However, in the counterfactual some premature deaths in low-SES localities will be prevented, leading to an increase in total future health care costs on the patients whose life been extended. Hence, the health care system may see some *savings* in current disparities, like the government saves on old-age allowances due to premature deaths. Despite that, we don’t estimate the possible savings to the health care system. Such a calculation is much harder, and will require further data or assumptions, as future costs are not set by law (as in old-age allowances) but depend on the future health status of the patients whose life is extended. If these patients enjoy also low-morbidity in the extended life years, the additional costs to the system will not be as high.

We survey only the Ministry of Health’s expenditures on reducing health inequalities, but additional players in the healthcare system also spent large sums on such issues [30–33]. The HMOs, for example, implemented focused intervention programs and expanded health services in clinics in low socio-economic neighbourhoods, subsidized co-payments for the poor and so on. Health inequality leads to extra expenses that are not itemized here, also outside of the health care system. An example is the cost imposed on welfare bureaus for clients who cannot afford medicines and transport to medical care. According to a recent estimate by the Family Care Division of the Ministry of Social Services [34], 30% of all payouts to families by welfare-bureaus’ social workers are for health-related matters.

We find a large economic cost due to SES-related health inequalities. How can this burden be reduced? The MoH focuses mainly on reducing inequalities in access to medical care and on improving the health of disadvantaged population, taking the level of socioeconomic inequalities as given. However, as income inequality is very high in Israel, a significant reduction in the economic cost of SES-related health inequalities will most likely require also to reduce the SES inequalities themselves. This is a societal task, involving the labor markets, the education and the welfare systems, as well as the MoH. When evaluating policies that affect SES inequalities, policy makers should also consider the effect they might have on related health disparities and their economic burden. This work does not purport to present cost-benefit calculations of programs to tackle inequality. However, our estimates of the cost of the disparities may serve as a first step toward understanding the benefits side in future analyses of intervention programs.

## Abbreviations

CBS: Israel central bureau of statistics
GDP: Gross domestic product
MoH: Ministry of health
NII: National insurance institute
SES: Socioeconomic status

## Footnotes

1 The calculations were based on sixty-seven as men’s retirement age and sixty-two for women. The discount rate used was 3.5 percent

2 The employment rate was calculated here as the number of employees and self-employed in the locality divided by the population aged 20–64.

3 The cost of a one-day absence is calculated by dividing the gross monthly wage by the number of work days in a month (22 days).

## Authors’ information

Eran Politzer is a PhD student in the Economics Track of the Health Policy Program at Harvard University. At the time of the study, Eran was an economist at the Bank of Israel’s Research Department.

Amir Shmueli is a Professor of Health Economics at the Hebrew University-Hadassah School of Public Health.

Shlomit Avni is the director of the Policy planning division in the Administration for Strategic and Economic Planning at the Israeli Ministry of health. The division is in charge, amongst other things, for thr reduction of health inequalities. She holds a PhD degree from The Department of Politics and Government in Ben-Gurion University of the Negev.
